# Biomarkers for immunotherapy in esophageal cancer

**DOI:** 10.3389/fimmu.2023.1117523

**Published:** 2023-05-01

**Authors:** Xuelian Wang, Ping Wang, Xiang Huang, Yanan Han, Pei Zhang

**Affiliations:** ^1^ Department of Oncology and Hematology, Zhongxian People’s Hospital, Chongqing, China; ^2^ Department of Urology, Zhongxian People’s Hospital, Chongqing, China; ^3^ Department of Radiation Oncology, The First Center of the Chinese PLA General Hospital, Beijing, China; ^4^ Department of Radiation Oncology, The Fifth Center of the Chinese PLA General Hospital, Beijing, China

**Keywords:** esophageal cancer, immunotherapy, biomarker, PD-1/PD-L1, cancer immunity

## Abstract

The development of immunotherapy, especially immune-checkpoint inhibitors targeting PD-1/PD-L1, has improved the outcomes of patients with esophageal cancer. However, not all population derives benefit from the agents. Recently, kinds of biomarkers were introduced to predict the response to immunotherapy. However, the effects of these reported biomarkers are controversial and many challenges remain. In this review, we aim to summarize the current clinical evidence and provide a comprehensive understanding of the reported biomarkers. We also discuss the limits of the present biomarkers and propose our own opinions on which viewers’ discretion are advised.

## Introduction

Esophageal cancer is the sixth leading cause of cancer-related death worldwide (1). The main histological types of esophageal cancer are esophageal squamous cell carcinoma (ESCC) and esophageal adenocarcinoma (EAC), and ESCC is the predominant subtype in Asia. The poor outcomes of patients with esophageal cancer attributed to the late diagnosis and a propensity for metastases (2). In recent years, the development of immunotherapy including adoptive cell transfer and immune checkpoint inhibitors (ICIs) has revolutionized cancer treatment (3–5). ICIs targeting programmed death receptor 1 (PD-1) and its ligand (PD-L1) have been proved to be promising in the treatment of kinds of cancers, also in esophageal cancer. However, only 20-40% of patients will benefit from ICIs and even fewer will have long-term disease control (6, 7). Therefore, it is critical to screen out population that will respond to these agents. Aim of this paper is to review established clinical evidence and provide current knowledge for biomarkers in esophageal cancer.

## PD-L1 status

PD-1, mostly expressed on tumor-infiltrating immune cells and PD-L1, expressed on antigen-presenting cells and tumor cells, could negatively regulate the antitumor immune response (8, 9). Using monoclonal antibodies to block PD-1/PD-L1 checkpoint axis restored antitumor immunity (10). As a result, it is reasonable to assume the expression level of PD-L1 as a biomarker to predict the efficacy of anti PD-1/PD-L1 antibodies. Actually, the predictive role of PD-L1 expression, which could be determined by immunohistochemistry (IHC), has been proved in kinds of cancer types based on current clinical evidence (11–14). PD-L1 expression could be assessed using different scoring algorithm including tumor cells and tumor-infiltrating immune cells. Tumor proportional score (TPS) is defined as the percentage of viable tumor cells with PD-L1 staining relative to all viable tumor in sample (15, 16). In non-small cell lung cancer cohort of KEYNOTE-001 trial, patients with advanced-stage received pembrolizumab, of those with a PD-L1 TPS > 50% had a response rate of 45.2%, versus 16.5% and 10.7% in patients with a TPS of 1-49% and < 1%, respectively (11). Combined positive score (CPS) was defined as the sum of all PD-L1 positive cells (including tumor cells, lymphocytes and macrophages) divided by all viable tumor cells×100 (17). Beyond the TPS and CPS scoring system, the PD-L1 tumor-infiltrating immune cell (IC) status was defined by the percentage of PD-L1-positive immune cells in the tumor microenvironment, which was seldom used in clinical trials (18).

Recently, the efficacy and safety of ICIs in esophageal cancer was established based on several phase III clinical trials. Although the predictive role of PD-L1 expression was analyzed, it was still controversial. In the KEYNOTE-590 trial (NCT03189719), pembrolizumab plus chemotherapy as first-line therapy for advanced or metastatic esophageal cancer was firstly evaluated. Combined treatment with pembrolizumab improved the PFS (HR=0.51, CI:0.41-0.65) and OS (HR=0.62, CI:0.49-0.78) in PD-L1 positive patients (CPS≥10). For patients with negative PD-L1 expression (CPS<10), the improved PFS and OS was not meaningful (19). Subsequently, the CheckMate-648, ORIENT-15, JUPITER-06 and ESCORT-1^ST^ evaluated the efficacy of different ICIs as first-line therapy in esophageal cancer (20–23). In these clinical trials, either TPS or CPS was determined and different cut-off values were set. Results showed that (as summarized in **Table 1**) ICI plus chemotherapy was superior to placebo plus chemotherapy for OS and PFS in patients with higher PD-L1 expression. However, in patients with negative or low PD-L1 expression, the benefits were not observed in all trials. In addition, it should be noted that most patients included in these trials were diagnosed with ESCC. Interestingly, when it comes to monotherapy of ICI, things seem different. In trial KEYNOTE-181, patients with advanced/metastatic esophageal cancer were assigned to pembrolizumab or chemotherapy. Final analysis showed OS was prolonged with pembrolizumab versus chemotherapy for patients with CPS ≥10 (median, 9.3 vs 6.7 months; HR=0.69, CI:0.52-0.93), and the OS benefit was not observed in patients with CPS <10 (24). The results were similar in trial ATTRACTION-3, ESCORT and RATIONALE-302, monotherapy of ICI in second-line treatment of esophageal cancer demonstrated no meaningful improvement in OS compared with chemotherapy in patients with negative PD-L1 expression (25–27). These results suggested that chemotherapy could induce immunogenic cell death of tumor and therefore increase the anti-tumor immunity, diluting the effect of PD-L1 expression. Therefore, combined therapy with ICI would be a better choice in patients with negative PD-L1 expression.

**Table 1:** PD-L1 status and immunotherapy efficacy in phase III clinical trials of esophageal cancer.

	CPS	TPS	PFS Hazard ratio (95%CI)	OS Hazard ratio (95%CI)
KN-590	≥10		0.51 (0.41–0.65)	0.62 (0.49-0.78)
	<10		0.80 (0.64–1.01)	0.86 (0.68-1.10)
ESCORT-1st		<1%	0.62 (0.46-0.83)	0.79 (0.57-1.11)
		≥1%	0.51 (0.39-0.67)	0.59 (0.43-0.80)
		<5%	0.60 (0.46-0.79)	0.77 (0.56-1.04)
		≥5%	0.52 (0.39-0.69)	0.60 (0.43-0.84)
		<10%	0.59 (0.46-0.75)	0.78 (0.59-1.02)
		≥10%	0.51 (0.36-0.72)	0.52 (0.35-0.79)
ORIENT-15	<10	<10%	0.53 (0.40-0.71)/CPS 0.56 (0.44-0.71)/TPS	0.62 (0.45-0.85)/CPS 0.67 (0.52-0.88)/TPS
	≥10	≥10%	0.58 (0.45-0.75)/CPS 0.54 (0.39-0.74)/TPS	0.64 (0.48-0.85)/CPS 0.55 (0.38-0.78)/TPS
	<5	<5%	0.51 (0.35-0.75)/CPS 0.57 (0.44-0.73)/TPS	0.56 (0.37-0.86)/CPS 0.61 (0.46-0.82)/TPS
	≥5	≥5%	0.58 (0.47-0.73)/CPS 0.54 (0.40-0.73)/TPS	0.65 (0.51-0.83)/CPS 0.67 (0.49-0.92)/TPS
	<1	<1%	0.76 (0.41-1.38)/CPS 0.52 (0.39-0.68)/TPS	1.32 (0.63-2.77)/CPS 0.55 (0.40-0.75)/TPS
	≥1	≥1%	0.54 (0.44-0.66)/CPS 0.59 (0.46-0.77)/TPS	0.59 (0.47-0.74)/CPS 0.71 (0.53-0.95)/TPS
JUPITER-06	<1		0.66 (0.37-1.19)	0.61 (0.30-1.25)
	≥1		0.58 (0.44-0.75)	0.61 (0.44-0.87)
	<10		0.56 (0.41-0.78)	0.61 (0.40-0.93)
	≥10		0.65 (0.45-0.92)	0.64 (0.40-1.03)
CK-648	<1	<1%	NA/CPS 0.95 (0.73-1.24)/TPS	0.98(0.50-1.95)/CPS 0.98 (0.76-1.28)/TPS
	≥1	≥1%	NA/CPS 0.65 (0.46-0.92)/TPS	0.69 (0.56-0.84)/CPS 0.54 (0.37-0.80)/TPS

CPS, combined positive score; TPS, tumor proportional score; PFS, progression free survival; OS, overall survival; NA, not applicable.

Generally, although high PD-L1 expression is evidentially associated with the efficacy of immunotherapy in esophageal cancer, it is still not perfect. The assessment of PD-L1 expression was usually made at the time of diagnosis or before treatment, which could not reflect the dynamic changes over time as the tumor microenvironment evolves. In addition, multiple treatments have been shown to induce the PD-L1 expression on tumor cells and antigen-presenting cells, such as radiotherapy, chemotherapy and targeted therapy (28–32). Also, the spatial heterogeneity of PD-L1 expression should be taken into consideration (33). As a result, the heterogeneity of PD-L1 expression makes it difficult to assess the PD-L1 status comprehensively and accurately, which could not be refined as a reliable biomarker when used alone.

## Tumor mutational burden

TMB is defined as the total number of mutations per coding area of a tumor genome, which is assessed by hybrid based next generation sequencing (NGS) (34). Somatic mutations in tumor have the potential to result in the expression of neoantigens, which could be recognized by the immune system and induce anti-tumor response (35). Based on this hypothesis, high TMB is thought to be correlated with benefits from ICIs administration. Current clinical evidence supporting TMB as a biomarker for immunotherapy come from trial KEYNOTE-158 (36). In this multi-cohort study, 1073 patients with previously treated advanced solid tumors were enrolled and efficacy was assessed in all patients who received at least one dose of pembrolizumab and had evaluable TMB data. The definition of TMB-high status was at least 10 mutations per megabase. Results showed that objective responses were observed in 30 (29%; 95%CI: 21-39) of 102 patients in the TMB-high group and 43 (6%; 5-8) of 688 in the non-TMB-high group, indicating TMB as a useful predictive biomarker for response to pembrolizumab monotherapy. Yarchoan and colleagues evaluated the relationship between TMB and objective response rate for immunotherapy. Through published researches, they plotted the objective response rate for anti-PD-1/PD-L1 therapy against the corresponding median TMB across multiple cancer types and observed a significant correlation (P<0.001) (37). Furthermore, the correlation coefficient of 0.74 suggested that 55% of the differences in the objective response rate across cancer types might be explained by TMB.

However, not all evidences are supportive. McGrail and colleagues collected clinical data of over 10000 patients from The Cancer Genome Atlas and analyzed the relationship between TMB and ICIs treatment outcomes. They found that in cancers where CD8 T cell levels positively correlated with neoantigen load, such as melanoma and lung cancer, high TMB predicted a better ORR to ICI significantly. However, in cancer types that exhibited no relationship between CD8 T cell levels and neoantigen load, such as breast cancer and glioma, high TMB showed poor clinical outcomes (38). In addition, esophageal cancer was classified as this type. These data did not support TMB as a predictive biomarker alone, other factors should also be taken into consideration. Yarchoan and colleagues assessed 9887 clinical samples for TMB and reported the median TMB across all samples was 3.48 mutations/Mb. Further analysis showed that the median TMB was less than 5 mutations/Mb and the percentage of a TMB greater that 10 mutations/Mb was about 10% in esophageal cancer (34). Based on these evidences, the predictive role of TMB for ICIs treatment is still controversial, especially in esophageal cancer. The use of TMB in clinical practice would be rare as the percentage of TMB-H is actually low in esophageal cancer. Also, we do not recommend TMB as a mono-biomarker.

## Microsatellite instability

Like TMB, MSI also represents the mutations in genome and is caused by defects in the mismatch repair (MMR) system, which leads to the production of neoantigens and further initiates antitumor response. Microsatellite is defined as repetitive DNA motifs of 1-6 base pairs, mainly occurring in non-coding DNA (39). The frequency of MSI differs among different cancer types. However, the available data was limited, except for colorectal cancer, gastric cancer and endometrial cancer for which MSI analysis was routinely performed. Bonneville and colleagues collected whole-exome data from 11139 tumor-normal pairs from TCGA and Therapeutically Applicable Research to Generate Effective Treatments projects and external data including 39 cancer types (40). Of all cancers analyzed, the frequency of MSI was 3.8% and MSI was not detected in 12 cancer types. Furthermore, the type-specific prevalence of MSI was analyzed, varying from 31.4% in endometrial carcinoma to 0.25% in glioblastoma. For esophageal cancer, MSI-H was observed in 3 (1.64%) of 184 cases, which ranked tenth of all types. Another study showed similar results. Parikh and colleagues tested tumor samples from 17486 unique patients with different cancer types and the prevalence of MSI-H was 1.7% (32/2501) in esophageal cancer (41).

Previous researches have shown that patients with MSI-H had a better response to immunotherapy. A phase 2 study (NCT01876511) enrolled 41 patients with progressive metastatic carcinoma treated with pembrolizumab. Data showed that the ORR and progression-free survival rate were 40% and 78% for mismatch repair-deficient colorectal cancer. In contrast, 0% of ORR and 11% of PFS rate were observed for mismatch repair-proficient colorectal cancers (42). In the cohort K of trial KEYNOTE-185, patients with MSI-H/dMMR advanced noncolorectal cancer were treated with pembrolizumab. Of all 233 enrolled patients (including 27 tumor types), 23 (9.9%) had a confirmed complete response and 57 (24.5%) had a confirmed partial response (43). These results supported MSI-H as a biomarker for immunotherapy.

However, the role of MSI status for esophageal cancer was not reported. In trial KEYNOTE-158, patients with MSI-H esophageal cancer were not included, probably resulting from the low prevalence of MSI in esophageal cancer. In trial KETNOTE-590, the MSI status was recorded for 112 (40%) of 278 patients and none had MSI-H (19). In addition, data of MSI status from other clinical trials for esophageal cancer was not available. Therefore, the predictive role of MSI status for immunotherapy in esophageal cancer requires further investigation and the conclusion is probably supportive by extrapolation from data for other cancer types.

## Nutritional status

The nutrition management draws more and more attention to date. In the natural course of cancer, poor nutritional status is prevalent resulting from the presence of the tumor and the patient’s treatment and is described as sarcopenia and cachexia. As a devastating and irreversible syndrome of energy imbalance, cachexia was reported to be responsible for the death of about 20% of all patients with cancer (44). Cachexia is characterized mainly as the loss of skeletal muscle, affecting 50%-80% of cancer patients. Recently, accumulating evidence emphasized the role of skeletal muscle cells in the regulation of immune response in health and disease (45, 46). Skeletal muscle cells express major histocompatibility complexes (MHCs) I and II under inflammation and are able to present antigens to T cells, acting as antigen-presenting cells. Furthermore, through secreting kinds of cytokines, skeletal muscle cells could regulate immune response (47). The most reported cytokine secreted by skeletal muscle cells is IL-15 (48, 49). IL-15 is critical in the development and maintenance of kinds of immune cells. The proliferation and activation of NK cells are regulated by IL-15. Also, the homeostasis of CD8 T cells and B cells are modulated by IL-15 (50). Based on these researches, skeletal muscle is thought to play a pivotal role in the interaction with immune cells, further regulating the immune responses. Therefore, more and more studies focused on the predictive role of skeletal muscle for the response to ICIs.

The condition characterized by the loss of muscle mass and strength in cancer patients is termed sarcopenia. In current studies, the skeletal muscle index (SMI) was mostly used to evaluate sarcopenia, which is defined as the ratio of skeletal muscle area (SMA) at the third lumbar vertebral level to squared height (51, 52). Roch and colleagues investigated the effect of sarcopenia on the efficacy of ICIs in non-small cell lung cancer. In this study, 142 patients were enrolled treated with first- or second-line ICIs and a decrease by 5% or more of SMI was defined as sarcopenia. Results showed that a shorter PFS and OS was observed in patients with sarcopenia, with HR: 2.45 (CI: 1.09-5.53) and 3.87 (CI: 1.60-9.34) respectively (53). Kim and colleagues also evaluated the prognostic significance of sarcopenia in patients with microsatellite-stable gastric cancer receiving ICIs. Of all enrolled 149 patients, 79 (53%) had sarcopenia. Patients with sarcopenia showed shorter PFS (1.4 moths vs. 2.6 months; P=0.026), and OS was shorter non-significantly (3.6 months vs. 4.9 months; P=0.052) (52). A meta-analysis was performed by Deng and nine cohort studies were included consisting of 740 patients with advanced cancer treated with ICIs. Data showed that patients with sarcopenia obtained a lower response rate than those without disease (30.5% vs. 15.9%; P=0.095). Further analysis found that sarcopenia was correlated with a significant shorter 1-y PFS rate (32% vs 10.8%; P<0.001) and 1-y OS rate (66% vs 43%; P<0.001) (54). Current evidence supported sarcopenia as a promising factor in patients receiving ICIs.

When it comes to esophageal cancer, nutrition management seems to be especially important. As is reported, the prevalence of sarcopenia in patients with esophageal cancer is high, ranging from 14.4% to 80% (55). Although the relationship between sarcopenia and the efficacy of ICIs in patients with esophageal cancer has not been fully studied, it is believed that sarcopenia would affect the efficacy of ICIs in patients with esophageal cancer extrapolating from data of other cancer types. Taking into consideration both the high prevalence and the extensive use of ICIs in esophageal cancer, it is necessary to reveal the prognostic value of sarcopenia.

## Inflammatory biomarkers

The relationship between inflammation and cancer is complicated. Tumor-associated inflammation is reported to promote cancer progression and even initiate the tumorigenesis (56). Current evidence revealed that the systemic inflammation is associated with the poor outcomes of cancer patients (57). However, the definition and quantification of systemic inflammation was not unified. C-reactive protein (CRP) and other combined factors were used in early studies, which has been proved to be prognostic markers in cancer patients (58). Recently, new associated factors were developed based on the measured full blood count, such as neutrophil to lymphocyte (NLR), derived NLR (dNLR), lymphocyte-monocyte ratio (LMR), platelet-lymphocyte ratio (PLR) or absolute count of white blood cell subtypes. These factors are inexpensive and easily obtained, indicating the potential use in clinical practice.

Of all the established factors, NLR was the most well-studied and shown to be predictive in survival across kinds of cancer types. Pirozzolo and colleagues conducted a meta-analysis and included 20 studies. In this study, 6457 patients with esophageal cancer were included and the role of NLR was analyzed, with the cut-off value ranging from 1.7 to 5. The HR for OS and PFS of all include studies was 1.6 and 1.66 respectively, indicating high NLR was related with poorer outcomes in esophageal cancer (59). Another study investigated the role of NLR to predict efficacy for ICIs treatment in patients with metastatic gastric cancer. In this study, best cut-off value was set as 3.23 and NLR < 3.23 was shown to be associated with longer OS (HR=0.38; CI: 0.26-0.57). Although NLR made no difference on ORR, NLR was still considered as a predictive factor for immunotherapy (60). Hwang and colleagues found that reductions in NLR were associated with the induction of IFN-γ responses that drove the expression of antigen presentation and proinflammatory genes sets. These results partially explained the underlying mechanisms of NLR prediction (61).

Evidence on the predictive role of inflammatory factors is accumulating and the value in esophageal cancer treated with chemoradiotherapy was also confirmed (62). However, it is unclear whether the inflammatory biomarkers are useful in patients with esophageal cancer receiving ICIs, which needs further investigation.

## Discussion

As the development of ICIs drugs, it is an urgent to introduce efficient biomarkers to improve the efficacy of immunotherapy. In trial IPASS (NCT00322452), 437 patients provided samples for EGFR mutation evaluation and 261 samples were positive for an EGFR mutation, of which 140 had exon 19 deletions and 111 had a mutation at exon 21 (L858R). The presence of a mutation of the EGFR gene was proved to be a strong biomarker of a better outcome with gefitinib (63). This research was a sort of opening salvo of the following development of targeted therapies. Though ICIs are targeting specific molecules as well, the molecules are not tumor driving. Therefore, the difference lies that ICIs exhibit anti-tumor effect not through specific molecules but by regulating the entire immunity. As is shown previously, a series of critical stepwise events must be initiated to develop anti-tumor immune response, which are referred as the Cancer-Immunity Cycle (64). Each of the biomarkers discussed in this review represents one or more of the steps in this cycle. For example, TMB and MSI-H refers to the release of cancer cell antigens and PD-L1 status is involved in the priming and activation of T cells. Absence of either event would lead to the arrest of the anti-tumor immune response. As a result, mono-biomarker is hardly able to predict the efficacy of immunotherapy. The author believes that a mathematic model covering all the pivotal seven steps must be established. By evaluating the anti-tumor immunity status of individual, physicians would make accurate judgements on the response of immunotherapy and provide more efficient treatment strategies. Although it is of great difficulty to establish such a model, the author considers it as a trend in the future development and interdisciplinary cooperation must be conducted.

## Author contributions

All authors contributed equally to drafting and writing the article. All authors revised it critically for important intellectual content. All authors read and approved the final manuscript.
